# The European Commission Consumers, Health, Agriculture and Food Executive Agency (CHAFEA) call for tender concerning studies on vaccination closes on 13 September

**DOI:** 10.2807/1560-7917.ES.2016.21.36.30337

**Published:** 2016-09-08

**Authors:** Eurosurveillance editorial team

**Affiliations:** 1 European Centre for Disease Prevention and Control (ECDC), Stockholm, Sweden

The European Commission Consumers, Health, Agriculture and Food Executive Agency (CHAFEA) call for tender to carry out two studies on the added value of strategic and life-course approach to vaccination, and on shortcomings related to low vaccination in healthcare workers, respectively, will close on 13 September 2016.

According to the tender document ‘each study has to include a representative number of Member States with a broad coverage as regards the variety of the governmental or constitutional structure/political concept, available legislative and managerial frameworks for programme coordination, vaccination programme performance, available programme capacities (infrastructure, human resources) within the EU.’

The contract, designed to carry out the two studies, is for EUR 220,000.000 and the duration is for 11 months.

Questions and answers for the tender were last updated on 31 August 2016.

Read more about the tender here .

